# Impact of the COVID-19 Pandemic on Community Pharmacy Services in New Zealand: A Repeated Cross-Sectional Best–Worst Scaling Analysis

**DOI:** 10.3390/pharmacy14020038

**Published:** 2026-03-02

**Authors:** Sepideh Sharif, Carla Dillon, Shane Scahill, Carlo Marra

**Affiliations:** 1 Faculty of Health and Sciences, Curtin University, P.O. Box U1987, Perth, WA 6845, Australia; sepi.sharif@curtin.edu.au; 2 School of Pharmacy, Memorial University of Newfoundland, 300 Prince Philip Drive, St. John’s, NL A1B 3V6, Canada; cmdillon@mun.ca; 3 School of Pharmacy, University of Auckland, 85 Park Road, Grafton, Auckland 1023, New Zealand; s.scahill@auckland.ac.nz

**Keywords:** consumer preferences, health service use, Best–Worst Scaling, pharmacy services, healthcare accessibility

## Abstract

Introduction: This repeated cross-sectional study examined community pharmacies in Aotearoa New Zealand and the services they provide, including retail, dispensing, and expanded scope services (e.g., minor ailment management). Methods: Two cross-sectional surveys were conducted in 2021 (n = 504) and 2023 (n = 1000). Both assessed demographics, service use, and perceptions of pharmacists. The 2021 survey focused on trust, approachability, and role awareness, while the 2023 survey added willingness to pay, telehealth use, and comparisons with other health professionals. Best–Worst Scaling and logistic regression quantified and compared preferences. Results: Prescription filling remained the most preferred service, while beauty product sales were least preferred. Preference for vaccination declined, indicating post-pandemic shifts in preventive care. Trust in pharmacists remained high, with strong comfort discussing health needs. Awareness of pharmacist roles improved slightly, though cost barriers persisted. Conclusions: Consumer priorities for prescription services remained stable, while interest in vaccination declined. The low preference for non-clinical retail activities suggests pharmacies should focus on health services. The high trust in pharmacists supports expanded clinical roles, but targeted policies and funding are needed to reduce cost barriers and enhance equitable access to primary healthcare.

## 1. Introduction

Community pharmacies in Aotearoa New Zealand (NZ) play a fundamental role in the healthcare system by acting as accessible hubs for medicine dispensing, minor ailment management, health education, and integrated care in collaboration with other healthcare providers. Unlike general practitioners or hospitals, pharmacies often serve as the first point of contact for individuals seeking healthcare advice, making them indispensable for improving public health outcomes and reducing the pressure on overstretched primary care services. However, despite their vital role, pharmacies operate within a fiscally constrained environment because many of the services they provide beyond prescription filling are not directly funded, leaving them reliant on dispensing fees and retail sales to sustain their operations [1].

The COVID-19 pandemic has accelerated these transformations, highlighting the resilience and vulnerability of community pharmacies [2]. As frontline healthcare providers, pharmacists played a crucial role in ensuring the continuity of care during lockdowns, distributing vaccines, providing health consultations, and managing increased public demand for accessible healthcare services [3]. The pandemic altered consumer behavior regarding pharmacy service utilization [4] and reshaped expectations regarding accessibility, affordability, and the range of services pharmacies should offer.

Recent policy reforms, such as the removal of prescription co-payments on 1 July 2023, aimed to reduce financial barriers to medicine access and reshaped consumer expectations [5]. Corporate and hybrid pharmacy models leveraged economies of scale to offer additional incentives, further influencing consumer preferences [6]. Understanding these evolving preferences can inform service enhancements, guide innovation, and strengthen the role of pharmacies in delivering patient-centered care.

Despite these shifts, empirical evidence on changing consumer preferences and public perceptions of pharmacists in New Zealand is limited [6,7,8]. Consumer preferences influence the uptake and effectiveness of community pharmacy services in primary care. Previous studies suggest pharmacists are seen as accessible prescribers, yet pharmacy-based services remain underutilized and inconsistently integrated into mainstream healthcare.

For clarity, pharmacy services can be conceptually categorized as Dispensing and Retail Services or Expanded Professional Services. Dispensing and Retail Services include routine activities such as dispensing medications, providing counseling at the time of dispensing, and selling over-the-counter or dermo-cosmetic products. Expanded Professional Services encompass extended clinical or health-focused activities that require additional competencies, training, or regulatory authorization, including vaccination, minor ailment management, point-of-care testing, and other specialized interventions. This distinction provides context for interpreting consumer preferences by recognizing differences in service complexity, professional requirements, and resource implications.

This study provides the first repeated cross-sectional analysis of how consumer preferences for pharmacy services shifted due to the COVID-19 pandemic. It highlights evolving public priorities in primary care access, informing how pharmacy services can be optimized for responsiveness and equity in future health policies.

The last major NZ studies of this type were conducted in 1988 [9] and 1990 [10]. Since then, pharmacy roles and expectations have changed substantially due to policy reform, funding models, and the impact of COVID-19. Overseas studies (e.g., in Canada, Ireland, and the UK) show the public generally views pharmacists as highly trustworthy professionals whose roles could expand to meet healthcare needs. For instance, an Irish study found that 85% of respondents believed pharmacies were good places to make more health services available, while, in Canada, 83% agreed pharmacists were underused in the healthcare system [11,12,13,14].

The Best–Worst Scaling (BWS) methodology was selected for this study because it allows respondents to identify the most and least important services within a set, providing more precise and discriminative insights into consumer priorities than traditional rating scales. This approach is particularly suitable for capturing nuanced shifts in preferences for community pharmacy services during and after the COVID-19 pandemic, as it reflects trade-offs that consumers make when choosing between multiple services.

Building on this evidence, the present study provides the first repeated cross-sectional analysis of how consumer preferences for community pharmacy services in New Zealand have shifted during and after the COVID-19 pandemic using Best–Worst Scaling surveys conducted in 2021 and 2023. It examines how evolving service use patterns relate to recent policy changes, such as the introduction of free prescriptions, and the growing influence of corporate and hybrid pharmacy models [6]. The findings aim to inform future pharmacy service planning, workforce development, and health policy to ensure accessibility and sustainability.

## 2. Methods

### 2.1. Survey Design

A literature search was conducted in PubMed (March–June 2020) for original research on public perceptions of pharmacists using the MeSH terms “Pharmacist,” “Public,” and “Perception.” Only English-language articles published from 2000 onward were included, as pharmacist roles have remained relatively stable during this period. Relevant studies [7,8,15] informed survey development.

Pharmacy organizations in countries with comparable practice environments, the United States, Australia, Poland, Ireland, the UK, and Canada, were contacted to access their survey instruments. Data from Poland, Ireland, the UK, and Canada were incorporated to enable cross-country comparisons of public trust and support for expanded pharmacy roles [11,12,13,14]. Historic NZ studies from 1988 [9] and 1990 [10] were also reviewed to ensure repeated cross-sectional continuity.

Attributes and survey items were developed collaboratively by the research team, a small convenience sample of practicing community pharmacists and health policy advisors, informed by the targeted literature review and previously published survey instruments. Attributes identified from these sources were reviewed iteratively by the research team and prioritized for inclusion based on relevance to the New Zealand healthcare context, feasibility within a Best–Worst Scaling (BWS) design, and their likely influence on consumer decision-making.

This formative qualitative work was undertaken prior to survey finalization to confirm the relevance, clarity, and completeness of the attribute list. Contributors were selected for their complementary expertise in pharmacy practice, consumer behaviors, and/or health service delivery.

This self-developed survey was piloted with a small convenience sample of 20 participants, and minor revisions were made to item wording and survey flow to improve clarity and usability prior to full deployment.

Content validity was ensured through expert review and iterative refinement. Formal psychometric validation (e.g., construct validity testing or internal consistency assessment) was not undertaken, as the questionnaire was designed to capture descriptive, population-level perceptions of pharmacy services rather than to function as a validated psychometric measurement scale. Demographic questions and BWS attributes were designed consistently across 2021 and 2023 surveys to enable valid cross-year comparisons.

### 2.2. Sample

The 2021 survey recruited 504 New Zealand adults, and the 2023 survey included 1000 participants nationwide. The 2021 survey was conducted during COVID-19, and the 2023 survey was conducted post-pandemic. The short timeframe allowed the assessment of changes in preferences due to policy changes, including the removal of prescription co-payments. In-person intercept sampling targeted a diverse cross-section of participants across age, sex, ethnicity, and region. Gender was documented, but no sex- or gender-based analyses were performed, as this was not the study focus. The two survey waves recruited independent samples; no participants were followed over time. Adults who had visited a pharmacy in the past 12 months were invited to participate via an intercept approach at public locations and community pharmacies. In 2021, data were collected onsite using paper-based questionnaires, which allowed the calculation of a response rate. In 2023, a mixed-mode approach combined onsite intercept recruitment with a QR code linking to an online survey, which did not allow the calculation of a response rate. Although recruitment locations and eligibility criteria were consistent across years, differences in data-collection modes may introduce selection bias, which should be considered when interpreting cross-year comparisons.

### 2.3. Data Collection and Measures

#### Survey Administration and Response Context

Surveys were administered using an in-person intercept approach at community pharmacy locations, with participants completing the questionnaire immediately following a pharmacy visit. While this approach facilitated the recruitment of a diverse national sample, responses may have been influenced by the immediate service environment or recent interactions with pharmacy staff. This potential source of response bias is acknowledged in the interpretation of findings.

Ethnicity was self-reported using predefined NZ census categories. Some survey services (e.g., Community Pharmacy Anticoagulant Management Service—INR monitoring for warfarin, methadone programs) involve specialized clinical knowledge, but were included to capture public awareness, perceptions, and acceptability of a broad range of pharmacy services.

A case 1 Best–Worst Scaling (BWS) experiment was conducted to identify the relative importance of pharmacy service attributes. A Balanced Incomplete Block Design (BIBD) was used to construct the choice tasks. Fifteen pharmacy service attributes were distributed across 15 choice sets, with seven attributes presented in each set. Participants were asked to select the most important (“Best”) and least important (“Worst”) service within each set. Each attribute appeared an equal number of times across the choice sets, ensuring design balance. Compared with traditional rating scales, BWS provides greater discrimination among attributes and reduces scale-use bias. The full BWS questionnaire, including all 15 choice sets, is provided in Supplementary Materials.

The 15 service attributes are:

1. Assessing and treating uncomplicated urinary tract infections (UTIs) and erectile dysfunction.
2. Blood checks (blood pressure, blood glucose).
3. Compounding medicines (e.g., creams).
4. Filling prescriptions and counseling patients on prescription medicines.
5. Receiving health advice from a pharmacist.
6. Infection testing (sexually transmitted infections, throat swabs).
7. Long-Term Condition (LTC) service (for patients with multiple chronic conditions).
8. Methadone program (supply and monitoring for opioid dependence).
9. Pharmacist supply of oral contraceptives (including emergency contraceptive pill and home pregnancy tests).
10. Sales of beauty products (e.g., makeup, perfume, sunscreen, ear piercings).
11. Sales of over-the-counter medicines (e.g., paracetamol, cold and flu products).
12. Smoking cessation services (nicotine cessation products, advice, referrals).
13. Travel services (passport photos, travel sickness medications).
14. Vaccinations.
15. Community Pharmacy Anticoagulant Management Service—INR monitoring for warfarin.

In 2021, only two perception measures were collected (trust in health advice, satisfaction after communicating with pharmacists). The 2023 survey included six measures (knowledge, satisfaction, comfort, encounters, essential role, advice-seeking). Overlapping 2021 measures were mapped to the 2023 survey for repeated cross-sectional comparisons. Survey items were structured using BWS methodology based on the prior literature. Multi-item scales for trust and satisfaction were piloted and demonstrated acceptable internal consistency (Cronbach’s alpha > 0.7), though they were not fully validated psychometric instruments.

Cross-year comparisons were limited to variables that were measured consistently across both survey waves, including demographic characteristics, BWS attributes, and overlapping perception items. Items that were included in only one survey year, or that differed in wording or response structure, were excluded from comparative analyses but remain reported descriptively for the relevant survey year.

### 2.4. Mixed Logit Model Specification

A mixed logit model was applied to estimate the relative importance of service attributes in consumer decision-making, incorporating both fixed and random coefficients. The utility function for each service is represented by the coefficient of each attribute (e.g., price, time, service), with the error term accounting for unobserved factors. Random coefficients were specified to capture preference variations across respondents, particularly for attributes like beauty product sales, which showed higher preference heterogeneity. The choice column in the dataset, based on the most and least preferred options, served as the dependent variable.

### 2.5. Model Estimation

The mixed logit model was estimated using the mixl package in R, utilizing 500 Halton draws to improve the estimation accuracy [16,17]. Maximum likelihood estimation was used, with a likelihood function incorporating random variations in preferences across respondents. Each participant’s choices across the 15 scenarios were modeled simultaneously, accounting for repeated measures.

### 2.6. Statistical Analysis

Means and standard deviations of coefficients indicated the direction and variability of preferences. Best–worst scores were calculated as the difference between the number of times an attribute was selected as best versus worst, standardized by the number of questions and respondents. Positive scores indicate stronger preference. The methodology followed established BWS guidelines [16,17]. Statistical significance was set at *p* < 0.05. Odds ratios were derived from significant coefficients to indicate relative likelihood of choosing each attribute. Demographic variables (age, gender, ethnicity) were included to explore consumer segment effects.

Differences between 2021 and 2023 survey samples were accounted for using mixed logit models with random coefficients and subgroup analyses to adjust for demographic and regional variations. Changes in preferences between surveys were analyzed by comparing Best–Worst scores and mixed logit model coefficients, with subgroup analyses and model-adjusted comparisons to account for demographic and regional differences.

**Figure 1:**
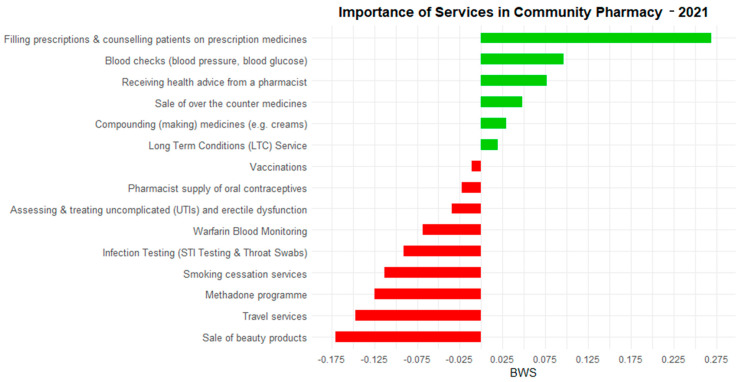
Relative preference for community pharmacy services in 2021. Green bars indicate services rated above the average (positive BWS) and red bars indicate services rated below the average (negative BWS) within the 2021 sample. Colors correspond to relative preference within this survey year. BWS scores are not intended for direct comparison with 2023 values.

**Figure 2:**
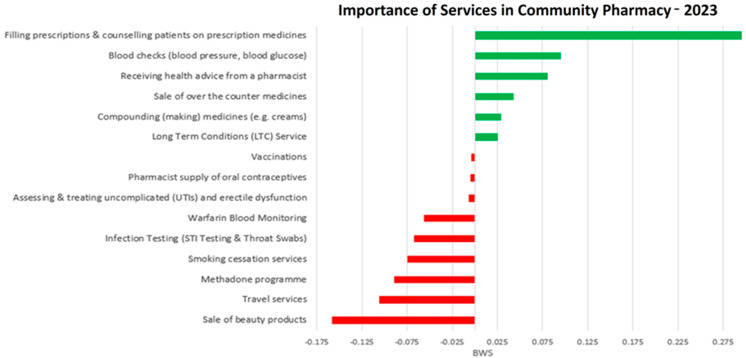
Relative preference for community pharmacy services in 2023. Green bars indicate services rated above the average (positive BWS) and red bars indicate services rated below the average (negative BWS) within the 2023 sample. Colors correspond to relative preference within this survey year.

To improve clarity in presenting survey results, BWS scores were visualized separately for each survey year. Figures were created to illustrate relative preferences within the 2021 and 2023 survey populations individually (Figure 1 and Figure 2). Green bars indicate services rated above the sample average (positive BWS), while red bars indicate services rated below the sample average (negative BWS). This approach ensures readability and avoids misinterpretation that could arise from combining the two survey waves. While trends across years can be interpreted qualitatively, BWS scores are relative to each survey sample and are not strictly comparable in absolute terms between 2021 and 2023.

## 3. Results

### 3.1. Survey Completions Pharmacy Use

In 2021, 504 New Zealand adults completed the survey (46.7% response rate), while the 2023 survey included 1000 valid responses nationwide (response rate not recorded). Both surveys used in-person intercept sampling in locations such as shopping centers and pharmacies to ensure demographic diversity across age, sex, ethnicity, and region (Table 1). The majority of participants self-reported their ethnicity as European (~60–70%), with the 2023 sample including a higher proportion of female respondents.

Table 1 includes age groups for both survey years and presents chi-squared test results for demographic comparisons. Age categories differ slightly from Table S1 in the supplementary because Table 1 applies harmonized age groupings required for valid statistical comparison across survey years, whereas Table S1 presents the original survey-specific age categories for descriptive completeness.

Readers should therefore interpret direct comparisons across tables with caution. It should be noted that age categories, as well as distributions of gender, education, and region, differed slightly between the 2021 and 2023 surveys; these differences may affect direct comparisons across years.

Most participants reported visiting franchised community pharmacies as their usual provider, with fewer indicating large chains or independent pharmacies (Supplementary Materials, Table S1). The majority reported visiting pharmacies at least once every 2–3 months, reflecting high baseline engagement. This frequent use highlights the accessibility and convenience of community pharmacies, particularly where general practitioner (GP) access may be limited or wait times are long. These findings are consistent with earlier national studies from 1988 and 1990 [9,10], emphasizing the potential for pharmacies to support public health initiatives and reduce pressure on other parts of the healthcare system.

### 3.2. Service Preferences and Importance Rankings

Figure 1 and Figure 2 present BWS scores separately for 2021 and 2023, illustrating the relative importance of pharmacy services within each survey population. Positive scores (green) indicate services rated above the average within that survey year, and negative scores (red) indicate services rated below the average. Presenting each year separately allows clear interpretation of the most and least preferred services within each population. Differences between years reflect trends in relative preferences rather than absolute changes in importance, as BWS scores are calculated independently within each survey sample.

Services such as beauty products, travel services, and smoking cessation were consistently ranked as least important. Between 2021 and 2023, the relative importance of core clinical services remained high, though there was some decline in strength, as reflected in odds ratios in Table 2. Table 2 is ordered by the absolute magnitude of change in logistic regression coefficients between 2021 and 2023 to facilitate comparison of shifts in consumer preferences.

### 3.3. Trust, Satisfaction, and Comfort with Health Professionals

**Figure 3:**
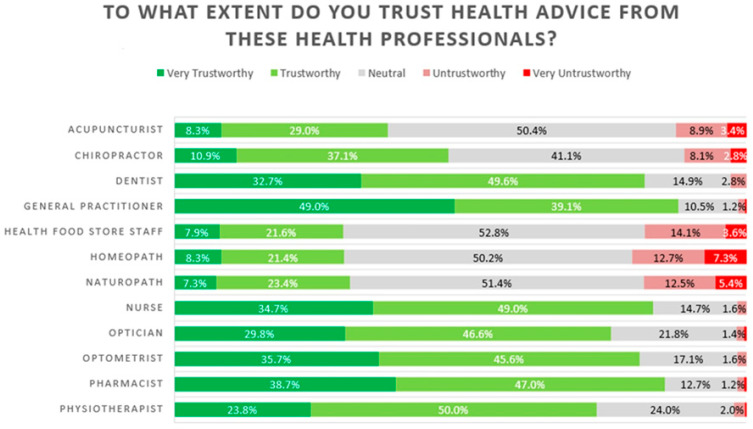
Trust in pharmacists, 2023.

Figure 3 shows consumer trust in health professionals in 2023 only. Trust and related perception measures were collected in 2023 to capture public confidence in pharmacists following the COVID-19 pandemic and recent policy changes; comparable data were not collected in 2021. Pharmacists were rated as highly trustworthy, comparable to nurses and slightly below general practitioners. Satisfaction after communicating with pharmacists was also generally positive.

Additional perception measures, including ratings of pharmacists’ knowledge, satisfaction, perception of their essential role, and comfort asking for health advice, are presented in Supplementary Figures S1–S4. These 2023-only measures provide additional context for post-pandemic trust in pharmacists. Detailed demographic subgroup analyses, and mixed logit model results are provided in Supplementary Tables S1 and S2.

### 3.4. Analysis of Preferences

The analysis revealed notable shifts in consumer preferences for pharmacy services between 2021 (Pharma survey, during-COVID-19) and 2023 (post-COVID-19 survey). Differences between survey years were assessed using chi-squared tests for categorical variables and z-tests for logistic regression coefficients.

Filling prescriptions and counseling (OR = 11.69, *p* < 0.001), blood checks (e.g., blood pressure, glucose monitoring) (OR = 4.32, *p* < 0.001), and receiving health advice from pharmacists (OR = 2.81, *p* < 0.001) were the most highly preferred services, as shown in Table 2, which is sorted by magnitude of change in preference between 2021 and 2023.

In the Best–Worst Scaling (BWS) analysis, participants selected only one best and one worst service per choice set. Consequently, the raw percentages reported in Supplementary Table S2 appear very low; these values represent selection probabilities and should be interpreted relative to other services rather than as absolute percentages of respondents.

After the COVID-19 period, these preferences remained among the top choices but were less pronounced, with odds ratios dropping to 3.68, 1.50, and 1.22, respectively (all *p* < 0.001). This suggests a general decline in the strength of preference for these core services, despite ongoing consumer interest.

Several other services showed a clear decline in preference between the two periods:

- Sale of beauty products: OR = 0.07 in 2021 vs. 0.04 in 2023.
- Smoking cessation services: OR = 0.35 vs. 0.32.
- Travel-related services: OR = 0.15 vs. 0.07 (all *p* < 0.001).

Although some differences reached statistical significance, the absolute magnitude of change for low-valued services—such as beauty products, smoking cessation, and travel services—was minimal. Because BWS scores are calculated independently within each survey sample, these comparisons reflect population-level trends rather than individual-level changes. Therefore, small shifts in low-priority services should be interpreted cautiously and as indicative of relative preference trends rather than absolute changes in importance. Services such as infection testing remained consistently low in preference, while long-term condition (LTC) services showed no significant change (*p* = 0.55). Logistic regression coefficients were tested against OR = 1 within each survey, with z-tests assessing differences between 2021 and 2023.

The mixed logit model revealed stronger positive effects for core clinical services post-COVID-19, reflecting heightened consumer reliance.

Preferences for beauty product sales and travel-related services were stronger during the COVID-19 period, while blood checks and LTC services remained stable. The alternative-specific constant was higher after COVID-19, indicating an overall increase in baseline preference for pharmacy services. Detailed results are provided in Supplementary Table S2.

## 4. Discussion

This study explores evolving consumer preferences for pharmacy services in Aotearoa New Zealand, highlighting a consistent prioritization of core clinical services such as prescription dispensing, blood pressure checks, and pharmacist-led health advice. These services remained valued over non-clinical offerings like beauty products or travel services, a trend consistent with previous findings. This aligns with previous findings that consumers see pharmacists as accessible health providers rather than retail staff [18,19].

International studies provide useful context for understanding public perceptions of pharmacists. For example, the Polish study [14] reported that the role of pharmacists is poorly understood by the public, with pharmacists often perceived primarily as dispensers of medicines. In contrast, the Irish study [12] found generally positive attitudes, highlighting community pharmacies as suitable venues for expanding health services. Similarly, the United Kingdom study [13] indicated high public trust in pharmacists’ health advice, and the Canadian study [11] suggested that a large portion of the public believes pharmacists are underused and supports expanding their role in healthcare delivery. These findings align with our results and provide an international perspective on evolving consumer expectations and trust in pharmacy services.

### 4.1. Key Findings and Interpretation

#### 4.1.1. Shifts in Service Priorities: Sustained Value of Core Clinical Offerings

The most striking result of the analysis was the decline in preference for vaccination services between 2021 and 2023, which likely reflects both behavioral and contextual changes over time. The high vaccination preference observed in 2021 occurred during the peak of the COVID-19 pandemic, a period characterized by heightened perceived risk, public health urgency, and limited access to general practitioners, which increased reliance on pharmacy-based vaccination. For example, in Italy, over two million COVID-19 vaccine doses were administered in community pharmacies following legislative changes enabling pharmacist-led vaccination [20,21]. By 2023, as COVID-19 restrictions eased and healthcare access normalized, vaccination services were perceived as less urgent, contributing to the observed decline. These changes reflect context-specific behaviors rather than long-term trends. Future research could explore vaccination preferences across pre-COVID, during-COVID, and post-COVID periods to more rigorously assess shifts in consumer behavior. Despite these contextual differences, consumers consistently ranked prescription filling, blood checks, and health advice as the most valued pharmacy services within each survey year (Figure 1 and Figure 2; Table 1). BWS scores are relative to each survey population; the observed differences between 2021 and 2023 indicate variations between the survey samples rather than definitive temporal changes in preference. Differences may partly reflect variations in sample composition rather than true changes in consumer priorities. This decline may also reflect broader availability of online or alternative healthcare providers and evolving healthcare behaviors post-pandemic.

The consistently low preference for beauty products underscores the limited appeal of non-healthcare-related offerings in pharmacies. This finding suggests pharmacies should prioritize healthcare-driven services and products to meet evolving consumer needs. However, the dual role of community pharmacies—as both retail businesses and healthcare providers—creates inherent tensions [22], as retail sales often subsidize unfunded clinical services, making it challenging to balance business sustainability with clinical service provision [18,19]. The observed shift in preference towards health consultations and OTC products further highlights the growing demand for professional health services, underscoring the need for careful planning and policy support to ensure both financial viability and high-quality clinical care.

#### 4.1.2. Dynamic Preferences Across Contexts

The mixed logit model results (Supplementary Table S2) shed light on how consumer preferences shifted in response to external factors such as the COVID-19 pandemic and policy changes. The results revealed a clear distinction in preferences between 2021 and 2023. Certain attributes, such as blood checks and UTI treatment, showed stronger preference weights after the COVID-19 period, reflecting a return to typical health concerns and routine clinical needs rather than pandemic-specific priorities. Conversely, services like methadone programs and oral contraceptive supply experienced modest positive shifts in preference under the modified scenario (post-policy change), while smoking cessation remained among the lowest-ranked services with a slight decline in preference, suggesting consumers placed greater value on public health and preventive services in this context.

These patterns are consistent with findings from other high-income countries with similar health systems. For example, studies in Canada and Ireland have found broad public support for expanded pharmacist roles and services [7,11,12]. Our finding that clinical services such as blood checks, UTI treatment, and health consultations remained valued, despite overall preference shifts, suggests alignment with this international trend.

Overall, these results underscore the dynamic and challenging role of community pharmacies in providing accessible clinical service provision whilst running sustainable businesses. These findings highlight the need for policy adjustments to ensure the sustainability of these services in the face of changing consumer expectations and market pressures [23,24].

#### 4.1.3. Public Perceptions of Pharmacists

High public trust and positive perceptions were evident in the survey. Figure 3 and Figures S1–S4 capture broader public sentiment toward pharmacists and pharmacy services. While trust (Figure 3) and satisfaction (Supplementary Figure S2) are comparable across 2021 and 2023, other perception measures—knowledge, encounters, essential role, and comfort asking for advice (Supplementary Figures S1, S3 and S4)—were only collected in 2023. These provide nuanced insights into post-pandemic attitudes but cannot be directly compared with 2021 data because BWS scoring and survey items differed between waves.

Trust levels remained exceptionally high, with pharmacists rated as highly trustworthy by over 80% of respondents, second only to general practitioners (Figure 3). Positive perceptions extended to pharmacist knowledge, service satisfaction, and comfort discussing health issues (Supplementary Figures S1–S4). The belief that pharmacists are essential healthcare professionals was also widely shared (Supplementary Figure S3).

### 4.2. Policy and Practice Implications

These findings suggest that pharmacy service development should prioritize clinical services where consumer trust and preference align. Sustained demand for core clinical services and high trust in pharmacists create opportunities for expanded roles in chronic disease management, preventive health services, and accessible healthcare delivery, particularly given New Zealand’s primary care capacity constraints.

### 4.3. Implications for Future Research

Future research should explore the drivers of changing service preferences and the public’s broader understanding of pharmacists’ roles, particularly in non-dispensing services.

## 5. Strengths and Limitations

It is important to note that the 2021 survey included substantial missing demographic data, and the overall composition of the 2021 and 2023 samples differed in terms of age, gender, and ethnicity (Supplementary Materials, Table S1). Observed differences between survey waves should be interpreted with caution, as variations in sample composition and contextual factors may partially explain apparent changes in preference patterns. The independent samples preclude definitive statements about temporal trends or preference decline. The absence of formal psychometric validation and the use of intercept sampling may have introduced measurement and response biases, which should be considered when interpreting the results.

A strength of our study is its ability to quantify changing consumer preferences over time using a robust BWS and mixed logit approach. However, we did not directly measure broader perceptions of pharmacist roles (e.g., understanding of scope), which previous NZ studies suggest remain important determinants of service use. Future research incorporating such measures would provide a more complete picture of consumer attitudes and inform effective service planning.

Some surveyed services, particularly specialized clinical programs such as Community Pharmacy Anticoagulant Management Service—INR monitoring for warfarin and methadone supply, may not have been fully understood by all participants, potentially influencing preference ratings. Additionally, societal stigma or biases, especially related to opioid dependence treatments, may have affected respondents’ evaluations. These factors represent limitations to interpreting consumer preferences and underscore the need for complementary input from healthcare professionals and enhanced public education to improve understanding and acceptance of such services.

## 6. Conclusions

This study highlighted the changing landscape of consumer preferences in the community pharmacy sector in Aotearoa, New Zealand. While pharmacies continue to play an essential role in providing healthcare services, evolving consumer expectations, especially post-COVID-19, indicate a need for adaptation. The growing preference for health consultations and essential healthcare services underscores the opportunity for pharmacies to enhance their role in public health. Policymakers should consider these findings when designing strategies to sustain pharmacy operations to ensure they remain responsive to both consumer needs and the broader healthcare environment. In addition to addressing evolving service preferences, strategies should leverage the high levels of public trust in pharmacists demonstrated in both historic NZ research and international studies. The effective communication of available services and expanded roles will be key to ensuring equitable access and optimizing the contribution of pharmacies to the wider healthcare system.

## Figures and Tables

**Table 1:** Demographic characteristics of participants and chi-squared test results for differences between 2021 and 2023 surveys. Shaded rows are used to distinguish section headers.

Demographic Variables	Survey (2023) (%)	Survey (2021) (%)
**Gender**		
Female	68.5	38.23
Male	31.5	34.54
Missing/Other	0.0	27.23
***p*** **-value**	<0.0001	
**Age Group**		
18–24	24.3	14.27
25–34	32.0	14.27
35–44	18.5	12.08
45–54	12.3	10.85
55–64	5.3	13.41
65–74	4.9	11.54
Missing/Other	2.7	23.58
***p*** **-value**	<0.0001	
**Ethnicity**		
European	50.0	53.45
Māori	21.0	5.87
Pacific	18.0	2.70
Asian	10.0	10.92
African	1.0	1.02
Missing/Other	0.0	26.04
***p*** **-value**	<0.0001	
**Region**		
Auckland	36.01	18.09
Hawke’s Bay	2.4	1.3
Canterbury	12.2	6.76
Bay of Plenty	7.3	2.32
Missing/Other	42.09	71.53
***p*** **-value**	<0.0001	

**Table 2:** Comparison of logistic regression coefficients for consumer preferences of pharmacy services during and after COVID-19.

Attribute	Survey (2021)	Survey (2023)
Filling prescriptions and counselling patients on prescription medicines	2.45	1.30
Blood checks (blood pressure, blood glucose)	1.46	0.40
Receiving health advice from a pharmacist	1.03	0.19
Sales of over-the-counter medicines (e.g., paracetamol, cold and flu products)	0.67	−0.21
Long-term condition (LTC) service (for patients who take multiple medicines for more than one chronic health condition)	0.52	−0.35
Compounding (making) medicines (e.g., creams)	0.49	−0.42
Vaccinations	0.01	−0.75
Pharmacist supply of oral contraceptives (including emergency contraceptive pill and home pregnancy tests)	−0.03	−0.29
Infection testing (sexually transmitted infection testing and throat swabs)	−1.059	−1.13
CPAMS: Community Pharmacy Anticoagulant Management Service—INR monitoring for warfarin	−1.46	−2.51
Methadone program—the supply and monitoring of methadone for opioid dependence	−1.57	−1.98
Smoking cessation services (e.g., pharmacist sale of nicotine patches and gum, advice, referral to support services)	−1.87	−2.45
Travel services (e.g., passport photos, sale of travel sickness medications and related products)	−1.90	−2.72
Sale of beauty products (e.g., makeup, perfume, sunscreen, including ear piercings)	−2.66	−3.24

## Data Availability

The data presented in this study are available on request from the corresponding author due to participant consent restrictions.

## Supplementary Materials

The following supporting information can be downloaded at: https://www.mdpi.com/article/10.3390/pharmacy14020038/s1, Table S1. Demographic characteristics of participants in 2021 and 2023 surveys (percentages among respondents with valid data). Table S2. The results of the mixed logit model, estimating the relative importance of service attributes in consumer decision-making. Figure S1. Rating pharmacist’s knowledge in 2023 surveys. Figure S2. Satisfaction after communicating with a pharmacist in 2023 surveys. Figure S3. How essential do you see the following health professionals in 2023 surveys. Figure S4. How comfortable do you feel asking each of these health professionals for health advice in 2023 surveys.
