# Supplementary material for: Impact of the COVID-19 Pandemic on Community Pharmacy Services in New Zealand: A Repeated Cross-Sectional Best–Worst Scaling Analysis
Source: Pharmacy (Basel). 2026 Mar 2;14(2):38. doi: 10.3390/pharmacy14020038 (PMC13010611; doi:10.3390/pharmacy14020038)
Supplement: Supplementary file 1 [file pharmacy-14-00038-s001.zip › pharmacy-4024869-supplementary.pdf]

Impact of the COVID-19 pandemic on community pharmacy services in New Zealand: a longitudinal best-worst scaling analysis

Sepideh Sharif [a], Carla Dillon [b], Shane Scahill [c], Carlo Marra*[,a]

[a] Faculty of Health and Sciences, Curtin University, Perth, Western Australia, Australia
[b] School of Pharmacy, Memorial University, Canada
[c] School of Pharmacy, Auckland University, New Zealand

*Corresponding author
Emails:
*carlo.marra@curtin.edu.au
sepi.sharif@curtin.edu.au
cmdillon@mun.ca.
s.scahill@auckland.ac.nz

[a] Postal Address: GPO Box U1987, Perth Western Australia, 6845, Australia
[b] Postal Address: School of Pharmacy, Memorial University of Newfoundland, 300 Prince Philip Drive, St. John's, NL A1B 3V6, Canada
[c] Postal Address: School of Pharmacy, University of Auckland, 85 Park Road, Grafton, Auckland 1023, New Zealand

ORCID: 0000-0002-3376-8169

Supplementary S. Supplementary tables and figures

This appendix provides supporting material referenced in the main text, including the complete survey questionnaire, full demographic data, extended statistical analyses (chi-squared and mixed logit models), and additional perception measures not shown in the main manuscript. The supplementary tables (S.1. & S.2.) and figures (S.1.–S.4.) expand upon the results presented in the main text.

Appendix Tables (S.1. & S.3.)

Table S.1.: It presents participants' age, gender, ethnicity, and pharmacy use characteristics for both survey years

Table S.2.: This table presents full mixed logit model coefficients and standard deviations for each service attribute, supporting the summary discussed in the Results section

Supplementary Figures (S.1.–S.4.)

Figure S.1.: Ratings of pharmacists' knowledge in 2023 surveys

Figure S.2.: Satisfaction after communicating with a pharmacist in 2023

Figure S.3.: Perceived essential role of pharmacists and other health professionals in 2023

Figure S.4.: Comfort level asking different health professionals for advice in 2023

Survey questionnaire

Table S.1.: Demographic characteristics of participants in 2021 and 2023 surveys (percentages among respondents with valid data)

**Variable**	**Category**	**2021 (%)**	**2023 (%)**
**Gender**	Male	48.8	31.0
	Female	50.6	68.5
	Gender Diverse/Other	0.6	0.5
**Age**	18–24 / 19–30	24.0	24.3
	25–34 / 31–40	18.1	32.0
	35–44 / 41–50	16.9	18.5
	45–54 / 51–60	17.3	12.3
	55–64 / 61–70	14.5	5.3
	65–74 / 71–80	7.9	4.9
	75+ / 81+	1.4	2.7
**Ethnicity**	European/Pākehā	71.6	60.5
	Māori	8.1	21.5
	Pacific Peoples	4.2	19.0
	Asian	17.5	10.0
	African / MELAA*	1.4	1.4
	Other	3.6	3.1
**Education**	Primary/Intermediate	3.4	5.3
	High School (NCEA or equivalent)	30.6	47.4
	Bachelor's/Diploma	41.7	34.9
	Postgraduate (Honours/Masters/Doctoral)	20.4	12.4
	Other/None	4.0	0.0
**Region**	Auckland	38.1	36.0
	Waikato	8.5	10.0
	Bay of Plenty	5.2	7.3
	Northland	4.2	2.9
	Canterbury	14.1	12.2
	Wellington / Capital & Coast	10.9	5.0
	Other DHBs combined**	-	-
**Rural**	Rural / Small towns	18.1	18.4
**Disability**	Yes	n/a	6.0
**Chronic condition**	Yes	n/a	22.5

*MELAA = Middle Eastern, Latin American, African

**2021 region categories varied; only major regions with >5% shown here for comparability. DHBs = District Health Boards

Note: Age categories differ between the two survey years, and other demographic variables (gender, education, region) also vary. These differences may affect direct comparisons across years.”

\

Table S.2.: Results of the mixed logit model estimating the relative importance of service attributes in consumer decision-making during Survey (2023) and Survey (2021) periods, including comparisons of attribute effects: Effect Comparison indicates whether each attribute had a stronger, weaker, or similar estimated impact on consumer choice during the Survey (2023) relative to the Survey (2021)

**Code**	**Attribute**	**Coefficients Survey (2023)**	**p-value Survey (2023)**	**Coefficients Survey (2021)**	**p-value Survey (2021)**	**Latitude effects (comparison)**
B_a1	Filling prescriptions	0.40	<0.01	0.58	<0.01	Stronger effect in Survey (2023)
B_a2	Blood checks	-0.32	<0.01	-0.33	<0.01	Similar effect in both
B_a3	Receiving health advice	0.50	<0.01	0.56	<0.01	Stronger effect in Survey (2023)
B_a4	Sale of over-the-counter	-0.27	<0.01	-0.26	<0.01	Similar effect in both
B_a5	LongTerm Conditions	0.17	0.01	0.06	0.52	Stronger in (2021)
ASC_B		2.82	<0.001	3.82	<0.01	Stronger in Survey (2023)
SIGMA_B		0.07	0.40	0.08	0.44	Similar effect in both
B_a6	Compounding medicines	0.05	0.40	0.02	0.81	Similar effect in both
B_a7	Vaccinations	-0.02	0.40	-0.08	0.09	Similar effect in both
B_Scenario_a1		0.002	0.65	-0.003	0.64	Similar in both
B_Scenario_a2		0.009	0.16	0.01	0.29	Similar in both
B_Scenario_a3		-0.015	0.03	-0.007	0.53	Stronger in Survey (2021)
B_Scenario_a4		0.007	0.05	0.002	0.65	Stronger in Survey (2021)
B_Scenario_a5		-0.012	0.10	-0.008	0.43	Similar effect in both
B_Scenario_a6		-0.006	0.31	-0.007	0.49	Similar effect in both
B_Scenario_a7		-0.001	0.54	0.000	0.91	Similar effect in both

Note: B_a1–B_a7 represent the main service attributes. B_Scenario_a1–B_Scenario_a7 represent scenario-specific effects capturing minor variations in choice sensitivity due to survey design or choice set context. Latitude effects indicate whether the effect of each variable was stronger in 2021 or 2023, or similar in both.

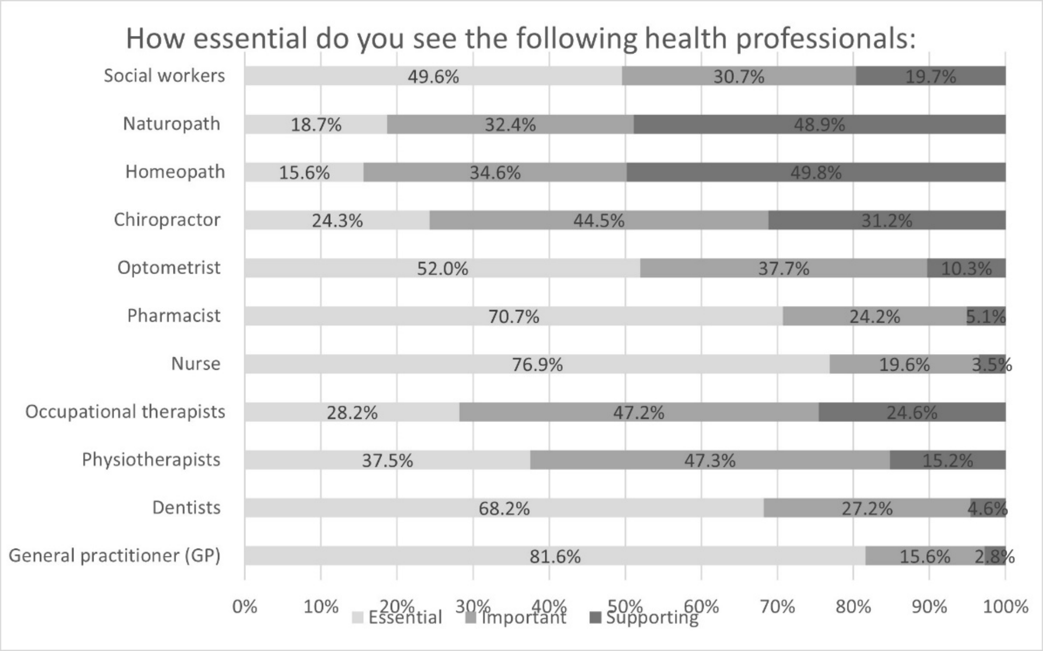

Figure S. 1.: Ratings of pharmacists' knowledge in 2023 surveys

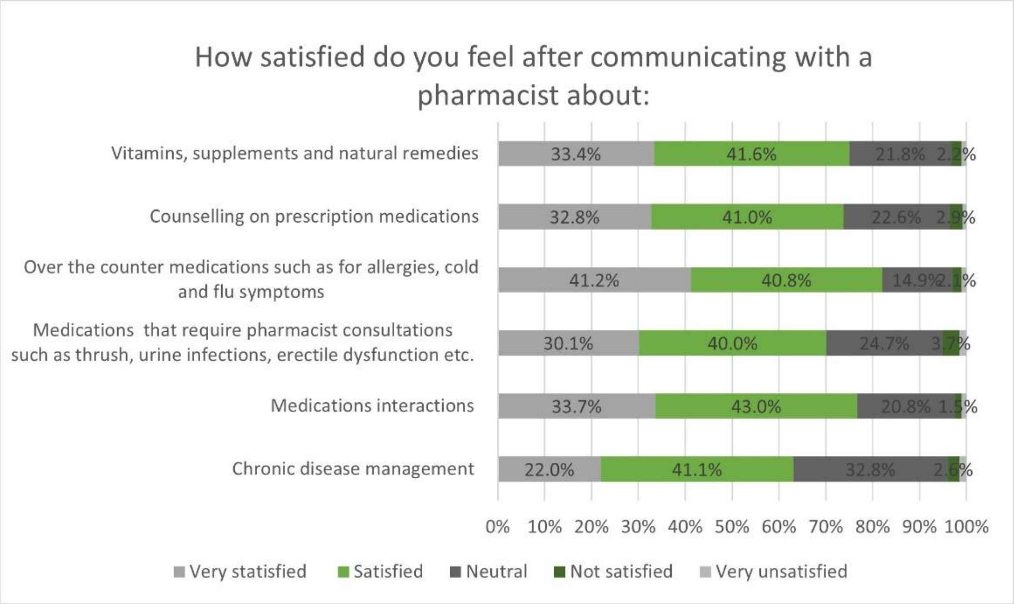

Figure S. 2.: Satisfaction after communicating with a pharmacist in 2023.

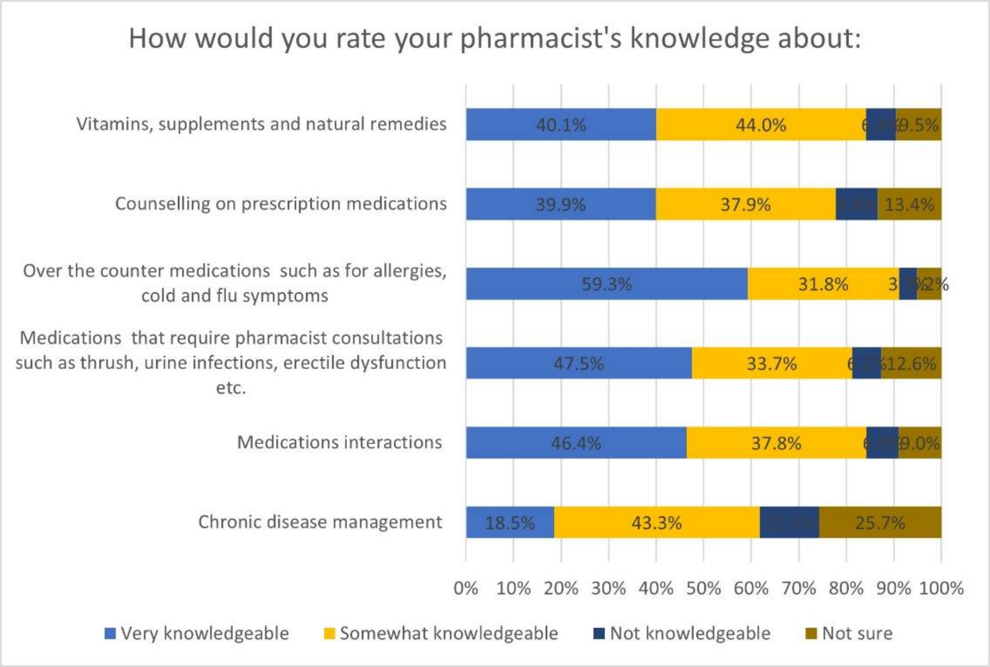

Figure S. 3.: Perceived essential role of pharmacists and other health professionals in 2023

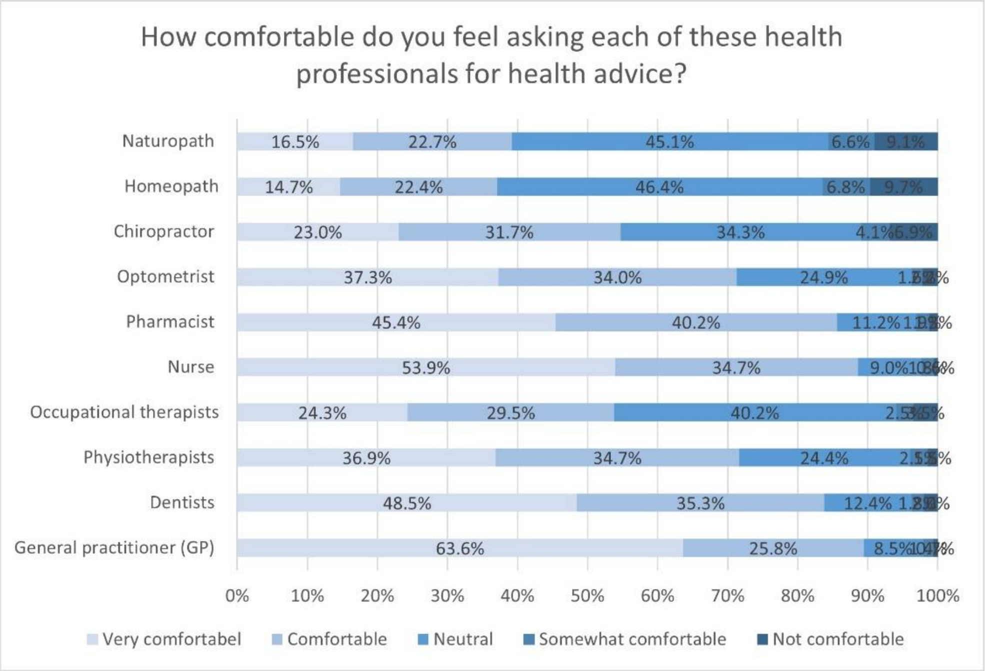

Figure S. 4.: Comfort level asking different health professionals for in 2023

Survey questions

Age:

- 18–24 years old
- 25–34 years old
- 35–44 years old
- 45–54 years old
- 55–64 years old
- 65–74 years old
- 75 and over

Gender (How do you identify as?):

- Male
- Female
- Gender diverse (please state)

Ethnicity (Tick as many as relevant):

- European/Pākehā
- Māori
- Pacific peoples
- Asian
- Middle Eastern/Latin American/African
- Other Ethnicity (please state)

What is the highest form of education you've received

- Primary school
- High school Diploma (NCEA or equivalent)
- Bachelor's Degree/Graduate Diploma
- Bachelor's Degree with Honours/Postgraduate Diploma
- Master's Degree
- Doctoral Degree

Which region in NZ do you live in?

- Northland
- Waitemata
- Auckland
- Counties Manukau
- Waikato
- Bay of Plenty
- Lakes
- Hauorā Tairāwhiti
- Taranaki

- Hawkes Bay
- Whanganui
- MidCentral
- Wairarapa
- Hutt Valley
- Capital and Coast
- Nelson Marlborough
- Canterbury
- South Canterbury
- Southern

Are you currently living in a rural area?

- Yes / No / Unsure

Do you identify as a disabled person?

- Yes / No

Do you have a chronic medical condition (e.g. diabetes, high blood pressure)?

- Yes / No

General questions

- To what extent do you agree with the following statements (Strongly agree, agree, disagree, strongly disagree):
- I understand what a pharmacist's role in health care includes.
- I am aware of most, if not all, of the possible health services my local pharmacy provides.
- My local pharmacy is more accessible than GP or nurse clinics.
- I trust the advice my pharmacist gives.
- The presence of gift items (such as soap, perfumes, makeup) in a pharmacy detracts from trust and confidence in the pharmacist profession.

How often do you visit your local pharmacy:

- Every day
- Every week
- Twice a month
- Once a month
- Every 3–4 months
- Every 6 months
- Once a year
- More than once a year

During COVID-19 pandemic restrictions (2020–2022), how often did you use telehealth to contact pharmacists?

- Every day
- Every week
- Twice a month
- Once a month
- Every 3–4 months
- Every 6 months
- Once a year

How likely will you use a telehealth consultation to contact pharmacists now in 2023?

- Very likely
- Likely
- Somewhat likely
- Not likely

How likely are you to use these types of pharmacies around NZ?

- Independent/Local
- Unichem
- Life Pharmacy
- Chemist Warehouse
- Bargain Chemist
- Countdown Pharmacy
- Pharmacy via Zoom or Telehealth

How would you rate the waiting times at your pharmacy for a prescription before the pandemic?

- Very efficient
- Acceptable
- Not very efficient
- N/A / cannot remember
- *How would you rate the waiting times at your pharmacy for a prescription now in 2023*?
- Very efficient
- Acceptable
- Not very efficient
- N/A / cannot remember
- Services provided

How important are these services? (Very important, Important, somewhat important, not important):

- Filling prescriptions
- Patient counselling on prescription medicine
- Blood checks (blood pressure, blood sugar)
- Sale of over-the-counter medicine

- Compounding medicines (e.g. creams)
- Vaccinations
- Pharmacist supply of oral contraceptives

Assessing and prescribing for common conditions (e.g. urine infections, eye infections, migraines, thrush, erectile dysfunction)

- INR testing (CPAMS: Community Pharmacy Anticoagulant Management Service – INR monitoring for warfarin)
- Infection testing (e.g. strep throat swab, RAT/COVID-19)
- Opioid Treatment Services
- Travel services (e.g. passport photos)
- Sale of beauty products

Which of these pharmacy services did you use most during COVID-19 restrictions (2020–2022)? (Tick as many as relevant):

- Filling prescriptions
- Patient counselling on prescription medicine
- Counselling for oral COVID-19 antivirals
- Blood checks
- Sale of over-the-counter medicines
- Compounding medicines
- Vaccinations (including COVID-19)
- Pharmacist supply of oral contraceptives
- Assessing and prescribing for common conditions
- INR testing
- Infection testing
- Opioid Treatment Services
- Travel services
- Sale of beauty products
- Telehealth consultations

If you needed these medical services how likely would you go to a pharmacy? (Very likely, likely, somewhat likely, not likely):

- Advice on current medicines
- Flu shot vaccination
- COVID-19 booster vaccination
- Other vaccinations
- Emergency contraception
- Medicines for common conditions
- Testing for infections
- Screening for medical conditions
- Blood checks

- Lifestyle advice

If you needed medical advice on these conditions, how likely would you go to a pharmacy as a first-line option? (Very likely, likely, somewhat likely, not likely):

- Cold/Flu symptoms
- Skin conditions
- Allergy symptoms
- Stomach symptoms
- Fungal infections
- Mild/moderate pain management

New Zealanders could benefit from these services in pharmacies (Strongly agree, agree, disagree, strongly disagree):

- Medicine reviews and assessments
- Ordering and reviewing lab tests
- Blood sugar and cholesterol testing
- Smoking cessation programmes
- Home medicines delivery
- Prescription reminder services
- Prescribing pharmacists for certain medicines
- What other services do you think New Zealanders could benefit from? (Free text option)

If you had to pay for these services out-of-pocket, how likely would you still use them? (Very likely, Likely, somewhat likely, not likely):

- Patient counselling
- Pharmacist prescribing of COVID-19 antivirals
- Blood checks
- Compounding medicines
- Vaccinations
- Assessing and prescribing for common conditions
- INR testing
- Infection testing
- Opioid Treatment Service
- Telehealth consultations

If lifelong vaccinations were funded and available at pharmacies, how likely would you use this service?

- Very likely
- Likely
- Somewhat likely
- Not very likely

If there was one thing you want to see more of from a pharmacy, what would it be? (Free text)

If there was one thing you want to see less of from a pharmacy, what would it be? (Free text)

Perceptions on pharmacists

How would you rate your pharmacist's knowledge about (Very knowledgeable, somewhat knowledgeable, not knowledgeable):

- Chronic disease management
- Medicine interactions
- Medicines that require pharmacist consultations
- Over-the-counter medicines
- Counselling on prescription medicines
- Vitamins, supplements and natural remedies

How comfortable do you feel communicating with a pharmacist about (Very comfortable, comfortable, neutral, not comfortable, very not comfortable):

- Chronic disease management
- Medicine interactions
- Medicines that require pharmacist consultations
- Over-the-counter medicines
- Counselling on prescription medicines
- Vitamins, supplements and natural remedies

How satisfied do you feel after communicating with a pharmacist about (Very satisfied, satisfied, neutral, not very satisfied, not satisfied, not applicable):

- Chronic disease management
- Medicine interactions
- Medicines that require pharmacist consultations
- Over-the-counter medicines
- Counselling on prescription medicines
- Vitamins, supplements and natural remedies

How effective do you believe pharmacists are at communicating health information that is easily understood?

- Extremely effective
- Effective
- Average
- Not effective
- Extremely not effective

Overall, how would you rate your encounters with a pharmacist:

- Very positive

- Positive
- Neutral
- Negative
- Very negative
- Healthcare professionals

How essential do you see the following health professionals? (Essential, Important, Supporting):

- General practitioner (GP)
- Dentist
- Physiotherapist
- Occupational therapist
- Nurse
- Pharmacist
- Optometrist
- Chiropractor
- Homeopath
- Naturopath
- Social worker

Based on your experience, how would you rate your encounters with these health professionals? (Very positive, positive, negative, very negative, not applicable):

- General practitioner (GP)
- Dentist
- Physiotherapist
- Occupational therapist
- Nurse
- Pharmacist
- Optometrist
- Chiropractor
- Homeopath
- Naturopath

To what extent do you trust health advice from these health professionals? (Very trustworthy, trustworthy, neutral, somewhat trustworthy, not trustworthy):

- General practitioner (GP)
- Dentist
- Physiotherapist
- Occupational therapist
- Nurse
- Pharmacist
- Optometrist

- Chiropractor
- Homeopath
- Naturopath

How comfortable do you feel asking these health professionals for health advice? (Very comfortable, comfortable, neutral, somewhat comfortable, not comfortable):

- General practitioner (GP)
- Dentist
- Physiotherapist
- Occupational therapist
- Nurse
- Pharmacist
- Optometrist
- Chiropractor
- Homeopath
- Naturopath
- COVID-19

Given the strain on our healthcare system caused by staff shortages, do you see a larger or smaller role for pharmacists in these areas? (Larger, about the same, smaller, not sure):

- Vaccinations
- Walk-in clinic services to assess and prescribe for common conditions
- Testing and lab services
- Contraception prescribing
- Chronic disease management
- Opioid treatment services

What do you think of these ideas? (Good idea, somewhat good idea, neither good nor bad, somewhat bad, Bad idea):

- More government funding for community-based healthcare services
- More government funding to expand services pharmacists can offer
- What do you think we could do to address the crisis facing NZ's healthcare system? (Free text option)
